# lncRNA SNHG5 Modulates Endometrial Cancer Progression via the miR-25-3p/BTG2 Axis

**DOI:** 10.1155/2019/7024675

**Published:** 2019-11-03

**Authors:** Shi Li, Yanan Shan, Xiaoya Li, Cong Zhang, Sisi Wei, Suli Dai, Riyang Zhao, Xiwa Zhao, Lianmei Zhao, Baoen Shan

**Affiliations:** ^1^Research Centre, The Fourth Hospital of Hebei Medical University, Shijiazhuang 050011, China; ^2^Department of Obstetrics and Gynecology, The Fourth Hospital of Hebei Medical University, Shijiazhuang 050011, China

## Abstract

Endometrial carcinoma (EC) is one of the most common malignancies of the female genital tract, although the mechanisms of EC initiation and development remain incompletely understood. In this study, we demonstrated that the noncoding RNA SNHG5 can inhibit the proliferation, migration, and invasion of EC cells by suppressing the expression of its putative target miR-25-3p. Overexpression of miR-25-3p significantly promoted the proliferation, migration, and invasion of EC cells. In addition, we showed that miR-25-3p represses the expression of BTG2 by directly binding to the 3′-UTR of BTG2 mRNA. Furthermore, increased miR-25-3p expression and decreased SNHG5 and BTG2 expression were observed in EC tissues, and the expression of SNHG5 was negatively correlated to that of miR-25-3p but positively correlated to that of BTG2. In summary, for the first time, we report that the SNHG5/miR-25-3p/BTG2 axis plays an important role in EC progression and is of great potential clinical significance for EC diagnosis and therapy.

## 1. Introduction

Endometrial carcinoma (EC) is one of the most common female malignancies that threaten health and life [1]. Based on histopathological and endocrine factors, EC is traditionally classified into two different types: estrogen-dependent type I and estrogen-independent type II [2, 3]. In general, type I ES had a favorable outcome, but once metastasis or recurrence occurs, EC has a poor prognosis, regardless of the type or stage [4]. Furthermore, the incidence of EC is increasing, highlighting the importance of investigating the underlying mechanisms of EC initiation.

The majority of the human genome comprises noncoding DNA, and their products noncoding RNAs, such as long ncRNAs (lncRNAs) and microRNAs (miRNAs), have been shown to be important regulators in biological processes [5]. In particular, lncRNAs have gained a great deal of attention due to their association with cancer development. lncRNAs are larger than 200 nucleotides and lack an open-reading frame to be translated into protein [6, 7]. These transcripts can participate in the regulation of specific genes through different molecular mechanisms in almost every stage of the expression process [8–14]. Recently, an emerging hypothesis that lncRNAs work as competing endogenous RNAs (ceRNAs) has been supported by numerous studies. The binding of miRNAs to the 3′-UTR of target mRNAs is known to result in the degradation or translational repression of the target gene. In this context, lncRNAs can act as a sponge to competitively interact with miRNAs and consequently sponge miRNA away from its target mRNAs [15], which lead to the overexpression of the target mRNA. Increasing numbers of studies have demonstrated that abnormal expression of lncRNA plays pivotal roles in tumorigenesis and represent promising targets for tumor diagnosis and treatment. The lncRNA SNHG5 is a transcript of small nucleolar RNA host gene 5 that has been reported to suppress gastric cancer cells by competing miR-32 with the mRNA of Kruppel-like factor-4 (KLF-4) [15, 16]. In hepatocellular carcinoma, SNHG5 has been shown to promote tumor cell progression by sponging miR-26a-5p and subsequently modulating the downstream target GSK3*β* [17]. Abnormal SNHG5 expression is also associated with other disease occurrence, such as osteoarthritis, osteosarcoma, and colorectal cancer [18–20]. Nevertheless, the clinical significance and molecular roles of SNHG5 in EC remain unclear.

In this study, we observed that SNHG5 was downregulated in EC tissues and reversed the malignant phenotypes of EC cells *in vitro*. A mechanistic study of SNHG5 activity revealed that SNHG5 can directly bind to miR-25-3p and abolish its suppressive function against its target BTG2. Thus, the SNHG5/miR-25-3p/BTG2 axis plays an important role in EC cells and clinical tissues, indicating its potential for applications in EC diagnosis and therapy.

## 2. Materials and Methods

### 2.1. Patient Tissue Specimens

All EC and noncancer tissues were obtained from EC patients and volunteer donors at the Fourth Hospital of Hebei Medical University. All Fresh tissue samples were immediately frozen in liquid nitrogen after surgical removal and stored at −80°C. All patients and their relatives signed informed consent for the use of samples, and this study was approved by the Fourth Hospital of Hebei Medical University.

### 2.2. Cell Culture and Transfection

The EC cell lines KLE and HEC-1-B were obtained from the Cell Culture Center, Chinese Academy of Medical Sciences (Beijing, China). All cell lines were cultured in DMEM (Thermo Fisher Scientific, Waltham, MA, USA) supplemented with 10% fetal bovine serum (FBS; Hyclone, Logan, UT, USA) and 100 mg/ml penicillin and streptomycin (Thermo Fisher Scientific) in an incubator at 37°C under an atmosphere with 5% CO_2_. The SNHG5 overexpression vector or the miR-25-3p mimic (Gene Pharma, Shanghai, China) was transfected into KLE and HEC-1-B cells using RNAiMax and Lipofectamine LTX with Plus Reagent (Thermo Fisher Scientific).

### 2.3. RNA Extraction and Real-Time Quantitative PCR

Total RNA was isolated from cells or tissues using the TRIzol reagent (Thermo Fisher Scientific) and then was reverse transcribed to cDNA using Moloney murine leukemia virus reverse transcriptase (Promega, Madison, WI, USA) following the manufacturer's instructions. Reverse transcription for miRNA detection was performed using special primers (Table 1). qRT-PCR was performed using SYBR Green PCR Kit (Takara Bio, Otsu, Japan) with the primers listed in Table 1 in a StepOne Real-Time PCR System (Thermo Fisher Scientific). The expression of specific genes was normalized to that of GAPDH or 18S rRNA, and U6 snRNA was used as an endogenous control to evaluate the expression of miRNAs.

### 2.4. Cell Proliferation Assays

Cell proliferation assays (MTS) were performed according to the manufacturer's instructions (Promega, Madison, WI, USA). All KLE and HEC-1-B cells were seeded into 96-well plates in triplicate and incubated for 24, 48, 72, and 96 h with 100 *μ*l DMEM supplemented with 10% FBS. At the specific time points, 20 *μ*l of MTS solution was added to the cells and incubated for 2 h at 37°C. Subsequently, the absorbance of each well was measured using a microplate reader at 492 nm.

### 2.5. Transwell Assays

Transwell chambers (Corning Costar, Cambridge, MA, USA) were placed in 24-well plates (8 mm pore size). 1 × 10^5^ KLE and HEC-1-B cells were seeded on the upper chamber containing DMEM without serum, while 0.6 ml medium supplemented with 10% fetal bovine serum (FBS) was added to the lower chamber. After incubation for 24 h, the cells on the upper chamber were removed, and cells that had migrated or invaded were fixed and stained with crystal violet. Subsequently, 5 random selected fields of the stained cells were counted using a microscope.

### 2.6. Colony Formation Assays

Plate colony formation assays were performed in triplicate to measure the proliferation ability of a single cell. After transfection, 500 cells treated with 0.3% soft agar were seeded into each well of a 6-well plate and incubated for approximately 2 weeks. Subsequently, the cells were washed and stained with 0.1% crystal violet, and the number of colonies was counted using a microscope.

### 2.7. Western Blot Analysis

Cells were harvested and lysed in lysis buffer on ice; after centrifugation, protein concentrations were measured using a BCA protein assay kit (Beyotime, Nanjing, China). Protein extracts were resolved by SDS-PAGE and transferred to PVDF membranes. The antibodies used for western blotting included anti-BTG2 (Proteintech) and peroxidase-conjugated anti-mouse or rabbit IgG (Cell Signaling Technology). The antigen–antibody reaction was visualized by an ECL assay (EMD; Millipore, Billerica, MA, USA).

### 2.8. Luciferase Assays

For the luciferase reporter assay, complementary sequences for miR-25-3p in the 3′-UTR of BTG2 mRNA were predicted using TargetScan, chemically synthesized and inserted upstream of the Renilla luciferase (Rluc) gene. Subsequently, the luciferase reporter plasmid or the empty vector was cotransfected into EC cells with miR-25-3p mimics in a 24-well plate. Luciferase activity was determined according to the manufacturer's protocol after transfection using the Dual-Luciferase Reporter Assay System (Promega). The Rluc activity was normalized to that of firefly luciferase activity and presented as the percentage of inhibition.

### 2.9. Immunohistochemical Analysis

Paraffin-embedded EC tissues were dewaxed in xylol and rehydrated in an alcohol gradient. Next, the sections were treated with hydrogen peroxide for 20 min. Then, a citrate antigen retrieval solution (containing EDTA, pH = 8.0) was used to treat the sections to expose the antigens on the surface of the tissue. Subsequently, goat serum was added to block the nonspecific binding sites, and the sections were incubated with primary antibodies in a moist box overnight at 4°C. The sensitizer and secondary antibodies were separately incubated with the tissue sections for 20 min each, after which the sections were stained using a DAB kit before dehydration.

## 3. Results

### 3.1. Analysis of SNHG5 Expression in Public Tumor Sequencing Databases

**Figure 1:**
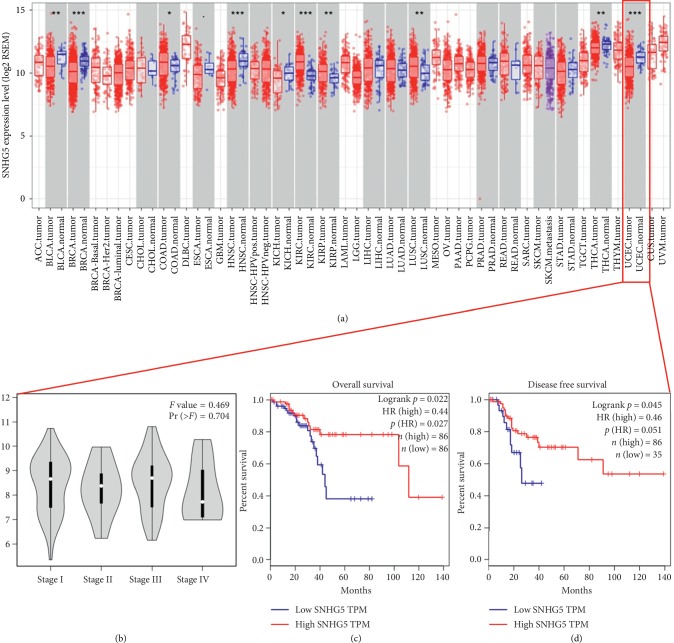
Expression of SNHG5 analyzed in the tumor sequencing public database. The RNA level of SNHG5 in different cancer types was analyzed using the public databases TIMER (https://cistrome.shinyapps.io/timer/) and GEPIA (http://gepia.cancer-pku.cn/). (a) SNHG5 was downregulated in EC. (b) SNHG5 showed the lowest level in stage IV compared to stages I–III. (c, d) EC patients with lower levels of SNHG5 showed a shorter survival time and disease-free survival times.

To investigate the clinical significance of SNHG5 in EC, we analyzed its expression using the public tumor sequencing databases GEPIA (http://gepia.cancer-pku.cn/) and TIMER (https://cistrome.shinyapps.io/timer/). The results of the analysis showed that SNHG5 exhibited significantly lower expression in EC tumor tissues compared to normal tissues (Figure 1(a)). Furthermore, in stage 4 tumors, a significantly lower level of SNHG5 was observed than in early-stage tumors (Figure 1(b)). In addition, survival analysis showed that the low expression of SNHG5 was a poor-prognostic factor that negatively correlated with the overall survival and disease-free survival of EC patients (Figure 1(c)). Taken together, these results suggest that SNHG5 is a molecular marker of EC.

### 3.2. SNHG5 Inhibits the Proliferation, Migration, and Invasion of KLE and HEC-1-B Cells

**Figure 2:**
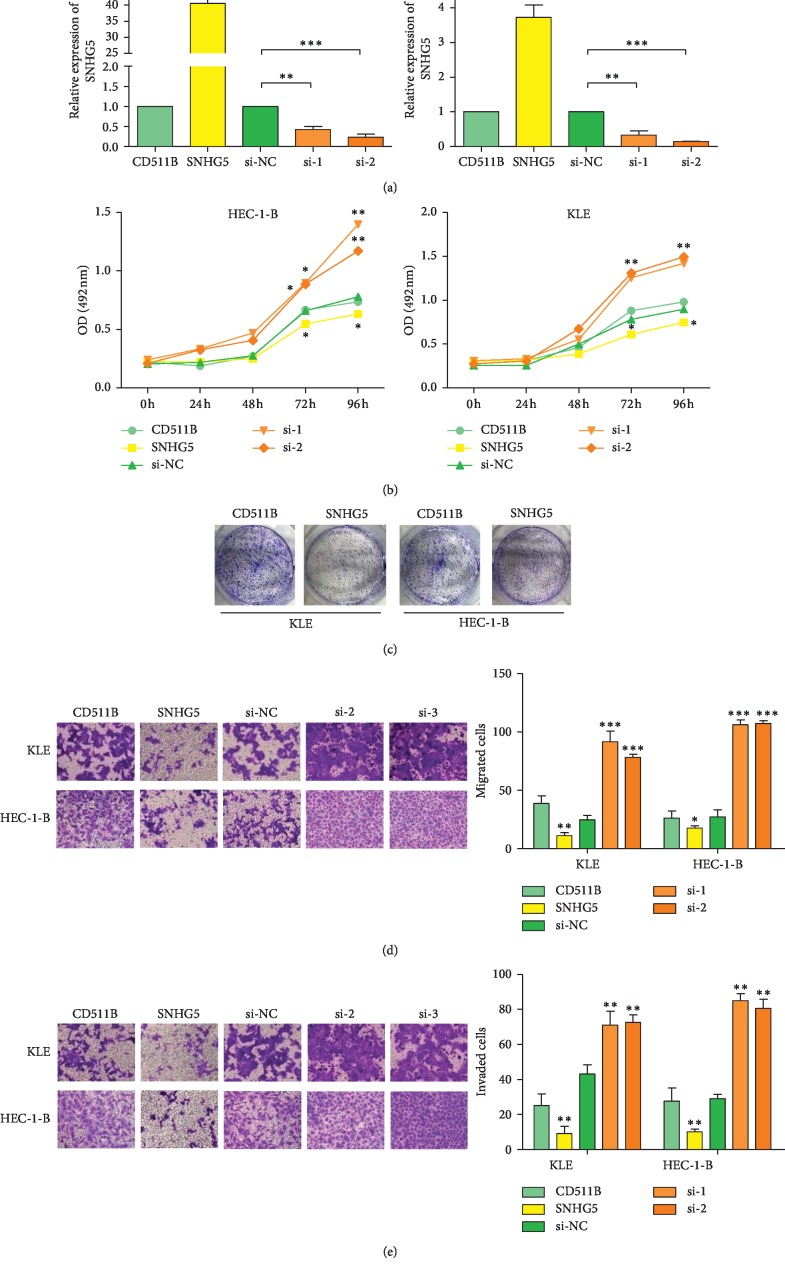
SNHG5 inhibits the proliferation, migration, and invasion of KLE and HEC-1-B cells. (a) Transcription analyses of SNHG5 in KLE and HEC-1-B cells after being overexpressed and knocked down. (b) Effects of SNHG5 overexpression and knockdown on cell proliferation analyzed by MTS assays. (c) Effects of SNHG5 overexpression and knockdown on the proliferation of a single cell analyzed by colony formation assays. (d) Effects of SNHG5 overexpression and knockdown on cell migration analyzed by transwell assays. Left: representative images are shown (×200). Right: normalized ratio of migrated cells. (e) Effects of SNHG5 overexpression and knockdown on cell invasion analyzed by transwell assays. Left: representative images are shown (×200). Right: normalized ratio of invaded cells. ^*∗*^*P* < 0.05; ^*∗∗*^*P* < 0.01; ^*∗∗∗*^*P* < 0.001.

To assess the effect of SNHG5 on EC development and progression, SNHG5 was knocked down by RNAi and overexpressed by transfection using a SNHG5 overexpression vector in both KLE and HEC-1-B cells. Transcription analyses showed that SNHG5 levels were reduced in SNHG5-siRNA transfected cells, whereas SNHG5 levels were increased in EC cells transfected with the SNHG5-overexpression vector (Figure 2(a)), demonstrating the success of genetic manipulation of SNHG5 expression in two EC cell lines. Subsequently, the molecular function of SNHG5 in KLE and HEC-1-B cells was further explored by MTS assays, colony formation assays, transwell migration assays, and matrigel invasion assays. The results showed that the proliferation of KLE and HEC-1-B cells was notably promoted by the inhibition of SNHG5 expression, whereas SNHG5 overexpression had the opposite effect (Figures 2(b) and 2(c)). Similarly, the migration and invasion abilities of EC cells also increased when SNHG5 was knocked down but decreased when SNHG5 was overexpressed (Figures 2(d) and 2(e)). These results indicated that SNHG5 is a tumor suppressor gene in EC cells.

### 3.3. SNHG5 Suppresses the Expression of miR-25-3p

**Figure 3:**
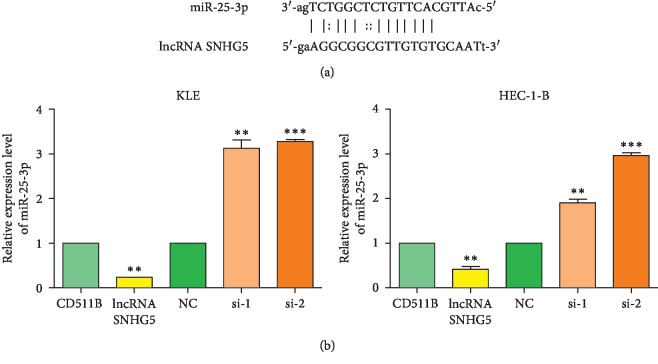
SNHG5 suppresses the expression of miR-25-3p. (a) Predicted binding site of SNHG5 with the putative target miR-25-3p. (b) Transcription analyses of miR-25-3p in KLE and HEC-1-B cells after being overexpressed or knocked down SNHG5. ^*∗∗*^*P* < 0.01; ^*∗∗∗*^*P* < 0.001.

Recent studies have demonstrated that lncRNAs can act as competing endogenous RNAs (ceRNAs) to directly bind specific microRNAs and decrease the production of mature miRNAs, leading to upregulated expression of miRNAs target genes. To elucidate the underlying mechanisms by which SNHG5 influences the development progression of EC, two microRNAs (miR-25-3p and miR-92a-3p) were predicted to be the binding targets of SNHG5 by the starBase, v2.0 program database (http://starbase.sysu.edu.cn). miR-25-3p has putative complementary sequences in SNHG5, indicating that SNHG5 may directly bind to and negatively regulate miR-25-3p expression (Figure 3(a)). We subsequently performed qRT-PCR to assess the expression of miR-25-3p in KLE and HEC-1-B cells after SNHG5 was overexpressed or knocked down. As expected, miR-25-3p expression was repressed after SNHG5 overexpression but was elevated in the SNHG5-knockdown KLE and HEC-1-B cells (Figure 3(b)). In contrast, the expression of miR-92a-3p was not normally suppressed by SNHG5 in these two EC cells (data not shown).

### 3.4. miR-25-3p Promotes the Proliferation, Migration, and Invasion of KLE and HEC-1-B Cells

**Figure 4:**
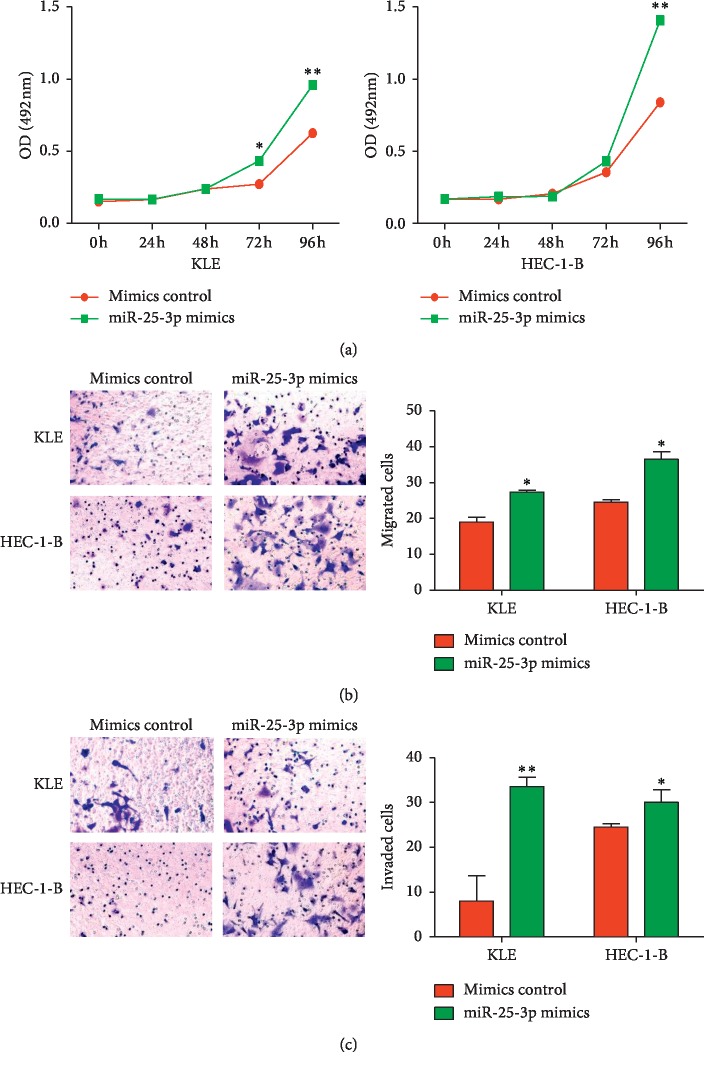
miR-25-3p promotes the proliferation, migration, and invasion of KLE and HEC-1-B cells. (a) Effects of miR-25-3p overexpression on cell proliferation analyzed by MTS assays. (b) Effects of miR-25-3p overexpression on cell migration analyzed by transwell assays. Left: representative images are shown (×200). Right: normalized ratio of migrated cells. (c) Effects of miR-25-3p overexpression on cell invasion analyzed by transwell assays. Left: representative images are shown (×200). Right: normalized ratio of invaded cells. ^*∗*^*P* < 0.05; ^*∗∗*^*P* < 0.01.

To determine whether miR-25-3p can affect EC development, miR-25-3p mimics were transfected into KLE and HEC-1-B cells. The results of MTS assays, transwell migration assays, and matrigel invasion assays suggested that overexpression of miR-25-3p accelerated the proliferation, migration, and invasion of KLE and HEC-1-B cells (Figure 4). These results demonstrated that miR-25-3p can promote the progression of EC cells.

### 3.5. SNHG5 Primarily Functions by Inhibiting miR-25-3p

**Figure 5:**
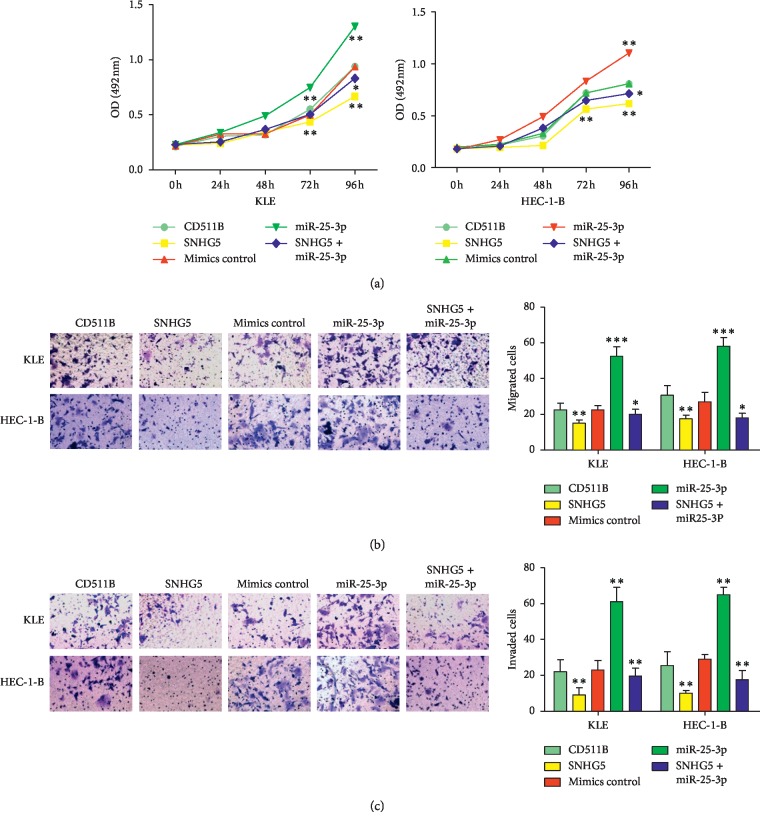
Rescue of SNHG5 function by overexpression of miR-25-3p. (a) Analyses of cell proliferation using MTS assays. (b) Analyses of cell migration using transwell assays. Left: representative images are shown (×200). Right: normalized ratio of migrated cells. (c) Analyses of cell invasion using transwell assays. Left: representative images are shown (×200). Right: normalized ratio of migrated cells. ^*∗*^*P* < 0.05; ^*∗∗*^*P* < 0.01; ^*∗∗∗*^*P* < 0.001.

SNHG5 and miR-25-3p have been shown to have opposite roles in KLE and HEC-1-B cells. To confirm whether SNHG5 suppresses the development of EC through its repression of miR-25-3p, miR-25-3p mimics were transfected into SNHG5-overexpressing KLE and HEC-1-B cells. Interestingly, the inhibition of KLE and HEC-1-B cell proliferation, migration, and invasion caused by SNHG5 overexpression was almost completely rescued by the transfection of miR-25-3p mimics (Figure 5). These results indicated that miR-25-3p is the central downstream target of SNHG5 in the suppression of EC cell progression.

### 3.6. BTG2 Is a Direct miR-25-3p Target

**Figure 6:**
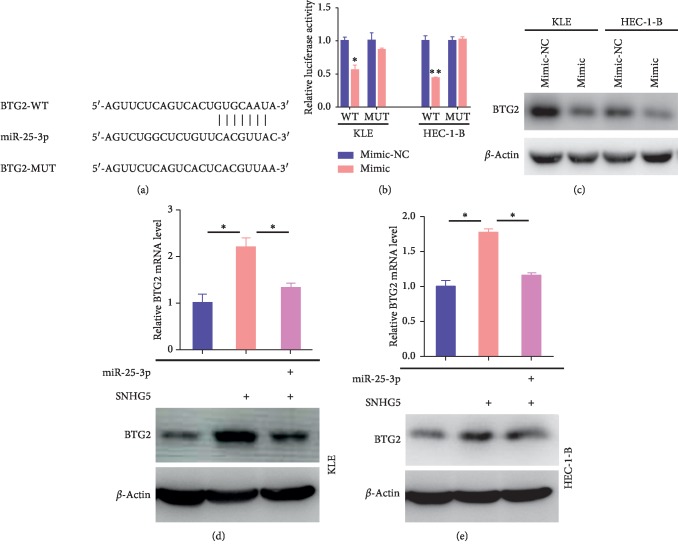
miR-25-3p directly suppresses its target BTG2. (a) Predicted binding site of miR-25-3p in the 3′-UTR of BTG2 and the corresponding mutant. (b) Luciferase activity analyses of luciferase regulated by the binding site of miR-25-3p or its mutant after miR-25-3p overexpression. (c) Western blot analysis of BTG2 after miR-25-3p overexpression. (d) Analyses of BTG2 expression detected by qRT-PCR (top) and western blot assays (bottom) in KLE cells after overexpressing SNHG5 or both SNHG5 and miR-25-3p. (e) Analyses of BTG2 expression detected by qRT-PCR (top) and western blot assays (bottom) in HEC-1-B cells after overexpressing SNHG5 or both SNHG5 and miR-25-3p. ^*∗*^*P* < 0.05; ^*∗∗*^*P* < 0.01.

Because miRNAs primarily function by directly binding to the 3′-UTRs of mRNAs and negatively regulate their stability and translation, we searched for potential targets of miR-25-3p using TargetScan (http://www.targetscan.org), the most commonly used software for miRNA target prediction. According to the predication results, B-cell translocation gene 2 (BTG2) was selected for further analysis due to the identification of an miR-25-3p binding site on the 3′-UTR of BTG2 mRNA, and reporter assays were performed to confirm this binding (Figure 6(a)). To construct the luciferase reporter system, the 3′-UTR of BTG2 mRNA or its mutant sequences at the putative binding site was cloned downstream of an Rluc reporter gene and then cotransfected with miR-25-3p mimics or a negative control into KLE and HEC-1-B cells. The results showed that the putative miR-25-3p binding sites notably decreased the luciferase activity in miR-25-3p-overexpressing KLE and HEC-1-B cells compared to the negative control. In contrast, the mutation of putative miR-25-3p binding site had no effect on the luciferase activity, indicating that miR-25-3p suppressed BTG2 expression by directly binding to the 3′-UTR of BTG2 mRNA (Figure 6(b)). In addition, the level of BTG2 protein was assessed by western blot assays. In agreement with the reporter assay results, BTG2 protein levels were decreased in KLE and HEC-1-B cells transfected with miR-25-3p mimics compared with those observed in the negative control (Figure 6(c)). These results firmly demonstrated that BTG2 is a direct target of miR-25-3p and that its expression is suppressed by miR-25-3p.

As mentioned earlier, SNHG5 can suppress the expression of miR-25-3p and BTG2 is a direct target of miR-25-3p. To determine whether SNHG5 can affect BTG2 expression through miR-25-3p, qRT-PCR and western blot assays were performed to analyze BTG2 expression in KLE and HEC-1-B cells after overexpressing SNHG5 or both SNHG5 and miR-25-3p. As expected, BTG2 expression was elevated by SNHG5 overexpression, whereas the elevated BTG2 expression decreased when we overexpressed SNHG5 while overexpressing miR-25-3p (Figures 6(d) and 6(e)). These results indicated that SNHG5 can increase the expression of BTG2 by inhibiting miR-25-3p expression.

### 3.7. Clinical Significance of the SNHG5/miR-25-3p/BTG2 Axis

**Figure 7:**
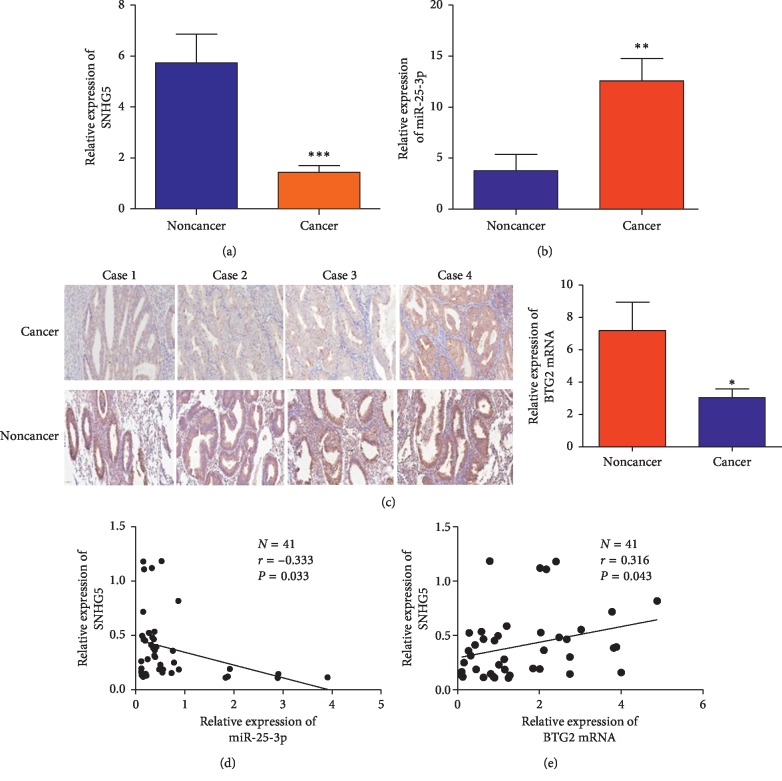
Clinical significance of the SNHG5/miR-25-3p/BTG2 axis. (a) Analyses of SNHG5 expression by qRT-PCR in clinical EC and noncancer tissues. (b) Analyses of miR-25-3p expression by qRT-PCR in clinical EC and noncancer tissues. (c) Analyses of BTG2 expression detected by immunohistochemistry and qRT-PCR in EC and noncancer tissues. ^*∗*^*P* < 0.05; ^*∗∗*^*P* < 0.01; ^*∗∗∗*^*P* < 0.001. (d) Negative correlation between the expression of SNHG5 and miR-25-3p in EC tissues. (e) Positive correlation between the expression of SNHG5 and BTG2 in EC tissues.

To assess whether the SNHG5/miR-25-3p/BTG2 axis plays a significant role in clinical samples, the expression profile of SNHG5 and miR-25-3p was investigated in 40 clinical EC tissues and 40 noncancer tissues. Consistent with the results in the public tumor sequencing database, SNHG5 expression was notably lower in EC tissues than that in noncancer tissues (Figure 7(a)). In contrast, the expression of miR-25-3p was upregulated in clinical EC tissues than that in noncancer tissues (Figure 7(b)). Moreover, a negative correlation between SNHG5 and miR-25-3p expression was observed by correlation analysis (*r* = −0.333; *P*=0.0332; Figure 7(d)), suggesting that the change in SNHG5 expression may induce abnormal production of mature miR-25-3p. These results demonstrated that there is a negative correlation between SNHG5 and miR-25-3p expression in EC tissues.

Considering that BTG2 is a target of miR-25-3p, we also analyzed the transcription profile of BTG2 in 40 EC tissues and 40 noncancer tissues by qRT-PCR, the results of which showed that the level of BTG2 mRNA was downregulated in EC tissues compared to that observed in noncancer tissues (Figure 7(c)). Furthermore, the expression of BTG2 protein was assessed by immunohistochemistry analysis in 49 EC and 20 noncancer tissues (Table 2), the results of which showed that BTG2 protein levels were notably decreased in EC tissues compared to the noncancer tissues (Figure 7(c)). In addition, the result of correlation analysis revealed a positive relationship between BTG2 and SNHG5 expression in EC tissues (*r* = −0.3163; *P*=0.0439) (Figure 7(e)). Taken together, these results demonstrated that the SNHG5/miR-25-3p/BTG2 axis is of great significance in EC cancer tissues.

In summary, SNHG5 inhibits EC cancer cell development by interacting with miR-25-3p, while miR-25-3p exhibits opposite effect on EC development by targeting BTG2 mRNA. Thus, the SNHG5/miR-25-3p/BTG2 axis might represent novel diagnosis and treatment targets for EC patients.

## 4. Discussion

EC is a highly malignant tumor that affects women worldwide. Although some molecular mechanisms involved in EC initiation and development have been elucidated in recent years, especially with respect to the effects of lncRNAs, such as MALAT1, linc-RoR, and H19, an understanding of EC progression is only partially complete [4, 21–23]. In this study, for the first time, we reported that the lncRNA SNHG5 can competitively interact with miRNA miR-25-3p to modulate the BTG2 protein expression in EC cells, providing new insights into the molecular basis of EC. Furthermore, the increasing incidence of EC and the lack of effective management strategies require additional tumor markers to be identified for diagnosis and treatment purposes. The abnormal expression of SNHG5, miR-25-3p, and BTG2 represents a new class of diagnostic markers for EC. Furthermore, the results of our molecular functional assays demonstrated that these molecules play important roles in maintaining the malignant phenotypes of EC cells and correlate with the progression and outcomes of EC patients. Thus, we also identified three potential promising intervention targets for therapies targeting EC.

SNHG5 is a snoRNA host gene that is also known as LINC00044. Both lncRNA SNHG5 encoded by its exons and snoRNA U50 and U50′ encoded by its introns have been reported to play important roles in different cancers. In our previously study, we reported that, in gastric cancer, SNHG5 is downregulated and functions in a dual manner. On the one hand, SNHG5 binds to the metastasis associated 1 family protein and modulated its cellular location [24]. On the other hand, SNHG5 can function as anmiR-32 sponge and promote the expression of the tumor suppressor KLF4 [15]. In this study, we showed that SNHG5 can also act as anmiR-25-3p sponge to modulate the expression of BTG2 and contribute to the progression of EC. In this respect, we not only identified a new downstream target of the tumor suppressor SNHG5 but also provided supporting evidence for the ceRNA regulatory network.

BTG family proteins, including BTG1, BTG2, BTG3, and BTG4, are structurally related proteins involved in cell proliferation. The results of previous studies have suggested that BTG family proteins play important roles in the regulation of the cell-cycle G1/S transition. When overexpressed, these BTG family proteins arrest cells in the G0 and G1 phases, which significantly inhibit cell proliferation [25–27]. Dysregulation of BTG2 expression has been previously reported in certain types of human cancers; nevertheless, its molecular functions in EC and its regulation remain poorly understood. In this study, we showed that BTG2 is a tumor suppressor that is downregulated in EC, expanding our understanding of BTG2. Furthermore, we demonstrated that BTG2 is regulated by the novel SNHG5/miR-25-3p axis. In this respect, these results provide important knowledge regarding the involvement of BTG family proteins in tumor development.

## 5. Conclusion

In summary, the results of this study increase our understanding of the molecular basis of EC progression and the regulatory functions of related molecules and identified promising diagnostic markers and therapeutic targets for EC. Furthermore, we will elucidate the function and mechanism of SNHG5 *in vivo* in the further study.

## Figures and Tables

**Table 1:** Primers used in this work.

Primers	Sequence (5′–3′)
*For reverse-transcription PCR*	
miR-25-3p-RT	GTCGTATCCAGTGCGTGTCGTGGAGTCGGCAATTGCACTGGATACGACTCAGAC
U6-RT	CGCTTCACGAATTTGCGTGTCAT

*For quantitative real-time PCR analysis*	
SNHG5-F	AAGCTTCTTTTACGTCGGCCTTCGCGAGCGTCTGG
SNHG5-R	GGATCCTCGAGTTAGTGGATTTTCCATTTAATGCTCC
miR-25-3p-F	GGCCATTGCACTTGTCTCGGTCTGA
miR-25-3p-R	CAGTGCGTGTCGTGGAGT
BTG2-F	CATCATCAGCAGGGTGGC
BTG2-R	CCCAATGCGGTAGGACAC
U6-F	GCTTCGGCAGCACATATACTAAAAT
U6-R	CAGTGCGTGTCGTGGAGT

**Table 2:** BTG2 expression level in EC and noncancer tissues.

Type of tissues	*N*	BTG2 expression		PR (%)	*P* value
		Low (+/++)	High (+++)		
Cancer	49	37	12	24.5	
Noncancer	20	6	14	70	0.001

*n*, number of total samples; PR, positive rate.

## Data Availability

All data supporting the conclusions of this work have been listed in this article.
